# Microglia are Required for Developmental Specification of AgRP Innervation in the Hypothalamus of Offspring Exposed to Maternal High Fat Diet During Lactation

**DOI:** 10.1101/2024.08.12.607566

**Published:** 2024-08-12

**Authors:** Haley N. Mendoza-Romero, Jessica E. Biddinger, Michelle N. Bedenbaugh, Richard B. Simerly

**Affiliations:** Dept of Molecular Physiology & Biophysics, Vanderbilt University, Nashville, TN, 37232, USA

**Keywords:** Microglia, Neural Development, Hypothalamus, Agouti-related peptide, Paraventricular hypothalamic nucleus, Major category, Developmental Biology, Minor category, Neuroscience

## Abstract

Nutritional fluctuations that occur early in life dictate metabolic adaptations that will affect susceptibility to weight gain and obesity later in life. The postnatal period in mice represents a time of dynamic changes in hypothalamic development and maternal consumption of a high fat diet during the lactation period (MHFD) changes the composition of milk and leads to enhanced susceptibility to obesity in offspring. Agouti-related peptide (AgRP) neurons in the arcuate nucleus of the hypothalamus (ARH) react to changes in multiple metabolic signals and distribute neuroendocrine information to other brain regions, such as the paraventricular hypothalamic nucleus (PVH), which is known to integrate a variety of signals that regulate body weight. Development of neural projections from AgRP neurons to the PVH occurs during the lactation period and these projections are reduced in MHFD offspring, but underlying developmental mechanisms remain largely unknown. Microglia are the resident immune cells of the central nervous system and are involved in refinement of neural connections and modulation of synaptic transmission. Because high fat diet exposure causes activation of microglia in adults, a similar activation may occur in offspring exposed to MHFD and play a role in sculpting hypothalamic feeding circuitry. Genetically targeted axonal labeling and immunohistochemistry were used to visualize AgRP axons and microglia in postnatal mice derived from MHFD dams and morphological changes quantified. The results demonstrate regionally localized changes to microglial morphology in the PVH of MHFD offspring that suggest enhanced surveillance activity and are temporally restricted to the period when AgRP neurons innervate the PVH. In addition, axon labeling experiments confirm a significant decrease in AgRP innervation of the PVH in MHFD offspring and provide direct evidence of synaptic pruning of AgRP inputs to the PVH. Microglial depletion with the Colony-stimulating factor 1 receptor inhibitor PLX5622 determined that the decrease in AgRP innervation observed in MHFD offspring is dependent on microglia, and that microglia are required for weight gain that emerges as early as weaning in offspring of MHFD dams. However, these changes do not appear to be dependent on the degree of microglial mediated synaptic pruning. Together, these findings suggest that microglia are activated by exposure to MHFD and interact directly with AgRP axons during development to permanently alter their density, with implications for developmental programming of metabolic phenotype.

## 1 Introduction

Maternal nutritional status has a profound effect on the metabolic phenotype of offspring. Children born to obese mothers experience higher rates of obesity later in life, with accompanying comorbidities that negatively impact health and longevity (Stettler et al., 2005; Prospective Studies Collaboration et al., 2009; Tamashiro and Moran 2010; Andersen et al., 2012). Although this developmental programming of metabolic phenotype has been reproduced in a number of animal models (Samuelsson et al., 2008; Masuyama et al., 2014; Garcia-Caceres et al., 2019; Skowronski, Leibel and Leduc, 2023), the underlying mechanisms remain poorly defined. In mouse models, maternal obesity during lactation (MHFD), a time when offspring are dependent on milk from their mothers for nutrition, appears to be particularly impactful. These changes to metabolic phenotype are thought to be mediated by changes in the milk (Gorski et al., 2006; Vogt et al., 2014; Calvo-Lerma et al., 2022) and occur without subsequent dietary challenge to the offspring themselves, suggesting that they are a consequence of developmental programming (Bolton et al., 2022; Skowronski, Leibel and LeDuc, 2023). Because neural circuits known to control body weight develop during the lactational period, they are vulnerable to a variety of environmental signals that may affect their organization and function (Horvath et al., 2010; Elson and Simerly 2015; Bouret, Levin and Ozanne, 2015; Zeltser, 2018; Skowronski, Leibel and Leduc, 2023).

AgRP neurons in the arcuate nucleus of the hypothalamus (ARH) function as “hunger neurons” that respond to key metabolic signals such as leptin, ghrelin, glucose and free fatty acids (Krashes et al., 2011; Betley et al.,2015; Chen et al., 2015; Sutton Hickey et al., 2023), and they distribute this information to other regions associated with energy balance regulation (Simerly 2008; Zagmutt, et al., 2018). Thus, the ability of AgRP neurons to influence other components of feeding circuitry is dependent on formation of their neural connections, which form primarily during the first 2 weeks of life (Bouret, Draper and Simerly, 2004a). During development, AgRP axons extend from the ARH at postnatal day 4 (P4) and reach the paraventricular hypothalamic nucleus (PVH) between P8-P10. Leptin is required for normal targeting of AgRP axons to their downstream targets, and in leptin deficient mice both neuroanatomical and related physiological defects persist into adulthood (Bouret et al., 2004b; Bouyer et al., 2013; Elson and Simerly 2015). Maternal overnutrition affects formation of feeding circuits during postnatal life with concomitant dysregulation of body weight (Plagemann et al., 1992; Elson and Simerly, 2015; Lippert and Bruning, 2022; Skowronski, Leibel and LeDuc, 2023). Limiting high fat diet exposure of dams to the first 3 weeks of lactation (MHFD) causes suppression of neural projections from AgRP neurons to the PVH in offspring and is associated with increased body weight later in life (Vogt et al., 2014). In fact, MHFD was more effective than prenatal maternal high fat diet exposure in causing body weight changes of adult offspring. MHFD did not alter cell number, peptidergic expression, or cellular activity of AgRP neurons in the ARH, suggesting that maternal nutritional status during lactation is particularly important for the establishment of neural connections related to the control of body weight. Notably, the effects of both leptin (Kamitakahara et al., 2018) and MHFD (Vogt et al., 2014) on targeting of AgRP projections display considerable regional specificity.

Adult mice placed on HFD display a marked hypothalamic gliosis that reveals an acute inflammatory response, which presages significant weight gain (Horvath et al., 2010; Valdearcos et al., 2017; Spencer et al., 2019; Cansell et al., 2021). This hypothalamic neuroinflammation is characterized by marked changes in the density and morphology of microglia that are most pronounced in the ARH (Thaler et al., 2012; Valdearcos et al., 2014). Microglia are the resident myeloid cells of the CNS and respond to a broad array of circulating signals, including nutrients such as saturated fats and carbohydrates (Valdearcos et al., 2014; Nadjar, et al., 2017; Leyroll, Laye & Nadjar, 2019; Butler et al., 2020). Moreover, activation of microglia alone is sufficient to stimulate food intake and promote weight gain in adult mice, and perturbations that block activation of microglia reduce the metabolic disruption associated with neuroinflammation (Valdearcos et al., 2017; Rosin et al., 2018; Sun et al., 2023). Because of their established role as nutrient-sensing sentinels of hypothalamic neuroinflammation, and their documented participation in neural development (Stevens et al., 2007; Schafer et al., 2012; Stephan et al., 2012), microglia have been proposed as possible mediators of developmental programming caused by nutritional perturbations (Rosin and Kurrasch, 2019; Folick et al., 2021).

Evidence from several of lines of investigation indicate that microglia have multiple roles in brain development (Schwarz and Bilbo 2009; Tremblay et al., 2011; Miyamoto et al., 2016; Li et al., 2019). Although microglia were initially thought to remain quiescent until activation by neuroinflammation, (Nakajima & Kohsaka, 2001; Nikolic & Tan, 2005) in vivo imaging experiments in the cerebral cortex demonstrated continual activity of their cellular processes, which actively survey their local environment (Stowell et al., 2018), including direct contact with axons and dendrites (Wake et al., 2009; Schafer et al., 2013). In addition to impacting neuronal number and initial formation of neural circuits, microglia are thought to play an important role in synaptic refinement through selective elimination of synapses, a process termed synaptic pruning (Paolicelli et al., 2011; Schafer et al., 2012; Hong, Dissing-Olesen & Stevens, 2016). Thus far, the majority of microglial developmental studies have focused on their role in cortical or hippocampal circuits. However, transcriptional profiling suggests a great deal of regional and temporal variation in microglial cell type and activity (Hammond et al., 2019; Masuda et al., 2020; Young et al., 2021), and the effects of dietary interventions have largely focused on the ARH. Here we used Iba1 immunostaining to visualize and measure morphological changes in microglial morphology in the PVH of offspring exposed to MHFD and compared these effects with those observed in the ARH and bed nuclei of the stria terminalis (BST), a limbic target of AgRP neurons. We also used genetically targeted axonal labeling of AgRP neurons to directly visualize cellular interactions between microglia and labeled AgRP terminals in the PVH and ARH to determine if MHFD stimulates synaptic pruning in these regions. The results demonstrate regionally specific changes to microglia in the PVH of MHFD offspring that are temporally restricted to the period when AgRP neurons innervate the PVH. In addition, the axon labeling experiments confirm a significant decrease in AgRP innervation of the PVH in MHFD offspring, and for the first time provide direct evidence of microglial-mediated synaptic pruning of AgRP terminals in the hypothalamus. Microglial depletion experiments determined that the significant decrease in AgRP innervation of the PVH observed in MHFD offspring requires normal densities of microglia, and that microglia are required for the weight gain seen in offspring of MHFD dams at weaning. However, we did not detect a significant effect of MHFD on the degree of synaptic pruning in the PVH, suggesting an alternative microglial signaling mechanism yet to be identified.

## 2 Results

### 2.1 Microglia exhibit morphological changes in the PVH in response to MHFD during postnatal development.

**Figure 1.**
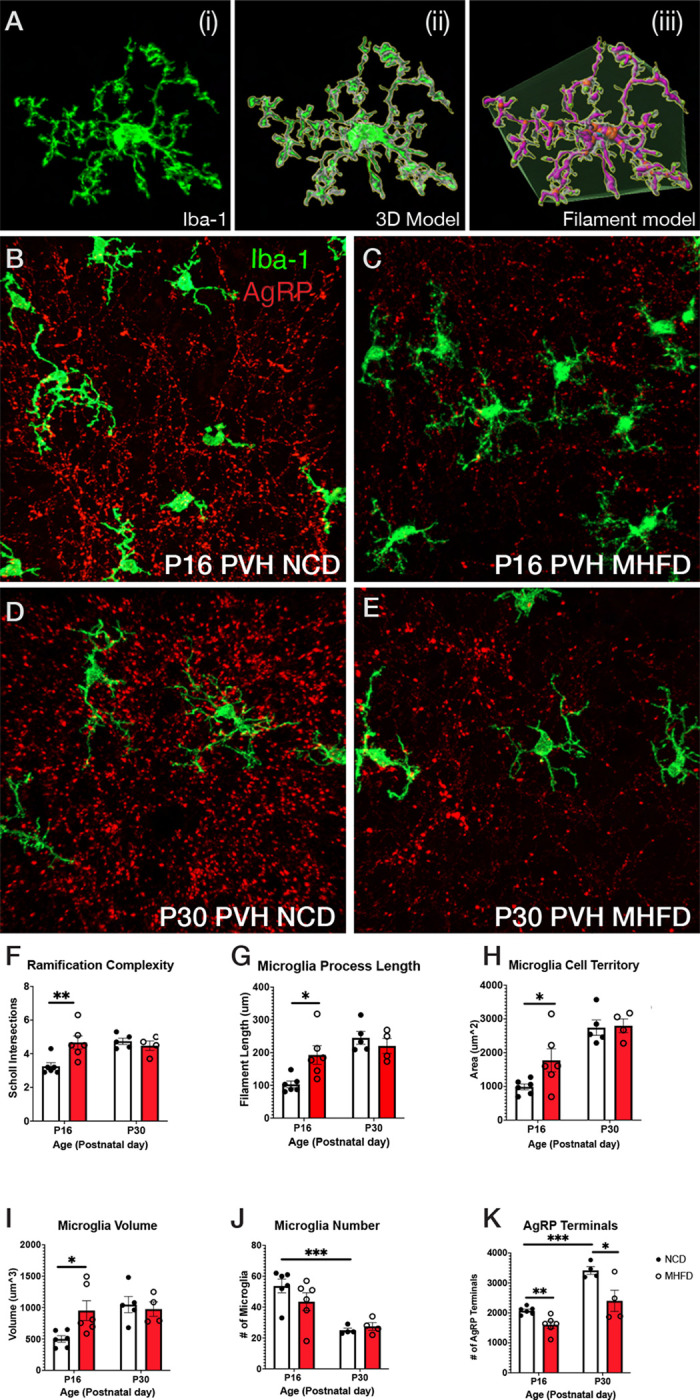
MHFD: Microglial morphology in the PVH. (A) Image analysis pipeline. Maximum projection of an Iba1 stained microglial cell in the PVH (Ai). Confocal images through labeled cells were used to generate 3D reconstructions (Aii). Each 3D rendering was then used to create a skeletonized model by using the Filaments tool in Imaris. Polyhedrons were generated around each cell using the Convex Hull function of Imaris to estimate the total tissue “territory” occupied by the microglial cell (Aiii). (B-E) Maximum projection images of microglial cells (green) and labeled AgRP terminals (red) in the PVH of mice at P16 (B,C) or P30 (D,E) that were either exposed to MHFD (C, E) or NCD (B, D). (F-J) Graphical comparisons between groups to show that MHFD increased microglial ramification complexity (F), microglial cell territory (G), cell volume (H) and process length (I) and AgRP terminals (K). The density of microglia in the PVH decreased between P16 and P30, irrespective of diet (J). Bars represent the mean ± SEM and each point represents one animal. *P<.05, **P<.005. Abbreviations: MHFD, maternal high fat diet during lactation; NCD, Normal Chow Diet; PVH, paraventricular nucleus of the hypothalamus.

To assess the impact of MHFD on microglia in the brains of postnatal mice, we used Iba1 immunohistochemistry and confocal microscopy to visualize the distribution and morphology of microglial cells in the PVH. Discrete regions of interest were imaged and a 3D modeling analysis pipeline was used to measure structural changes in microglia (Figure1Ai–iii). Although there was no apparent difference between the density of microglia in the PVH of MHFD and NCD offspring at P16 (Figure 1J), the overall size of microglia in the PVH of MHFD offspring was significantly greater, compared to that of NCD mice (Figure 1B, C). The enhanced size of microglia in the MHFD offspring corresponded to an 87% increase in overall process length and a 44% increase in branching complexity (Figure 1F, G). Additionally, the volume of microglial cells (volume of cell body and processes) and the spatial territory they occupy was nearly doubled in MHFD offspring (Figure 1H–I). These morphological changes appeared to be transient because both the density and size of microglia (process length and complexity) were reduced in postweaning mice perfused on P30 (Figure 1D–G). We also assessed the density of AgRP terminals in the same regions of interest in the PVH and confirmed that their density is significantly lower in the brains of MHFD offspring compared with that of NCD controls (Figure 1K), as suggested by data published previously (Vogt et al. 2014). Taken together, these results suggest that exposure to MHFD has a profound effect on the morphology of microglia that is consistent with enhanced activity and surveillance of their immediate microenvironment. Furthermore, the effects of MHFD on microglial morphology in the PVH of offspring display both temporal and regional specificity, which correspond to a decrease in the density of AgRP inputs to the PVH.

### 2.2 Microglia do not exhibit morphological changes in the ARH or BST in response to MHFD exposure during postnatal development.

**Figure 2.**
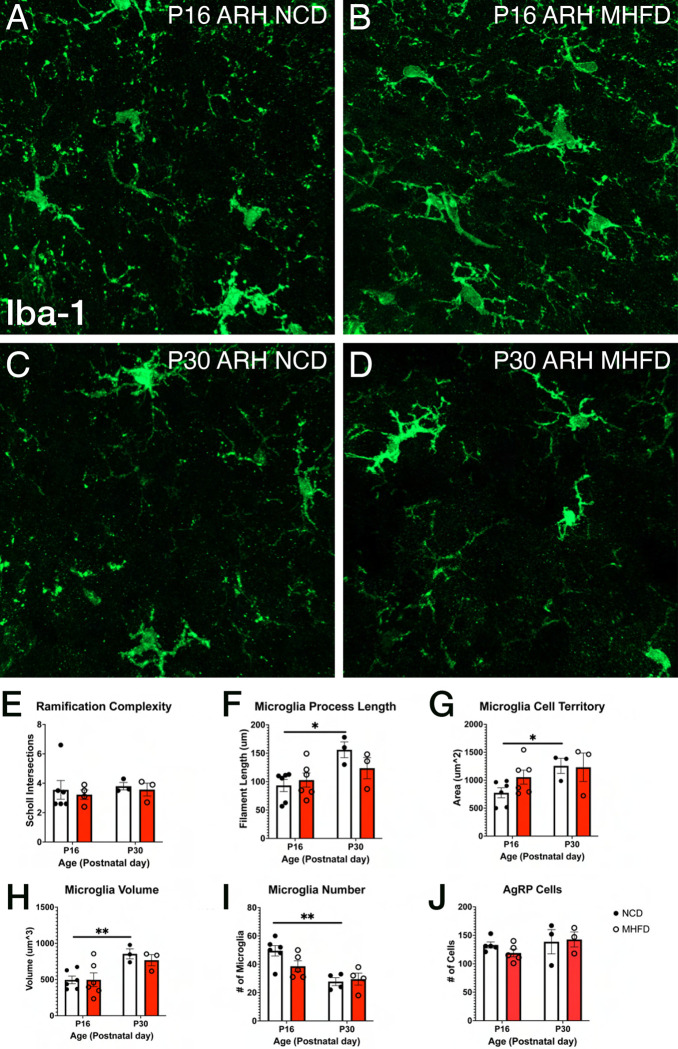
MHFD: Microglial morphology in the ARH. Maximum projection images of microglial cells (green) in the ARH of mice at P16 (A, B) or P30 (C,D) that were either exposed to MHFD (B, D) or NCD (A, C). Graphical comparisons between groups to show that microglial ramification complexity (E) remained the same, regardless of age or diet. Microglial cell territory (F), cell volume (G) and process length (H) increased between P16 and P30, but were not changed as a result of diet. The density of microglia in the ARH decreased between P16 and P30, irrespective of diet (I). There were no changes in numbers of AgRP neurons (J). Bars represent the mean ± SEM and each point represents one animal. *P<.05, **P<.005. Abbreviations: ARH, arcuate nucleus of the hypothalamus; MHFD, maternal high fat diet during lactation; NCD, Normal Chow Diet.

**Figure 3.**
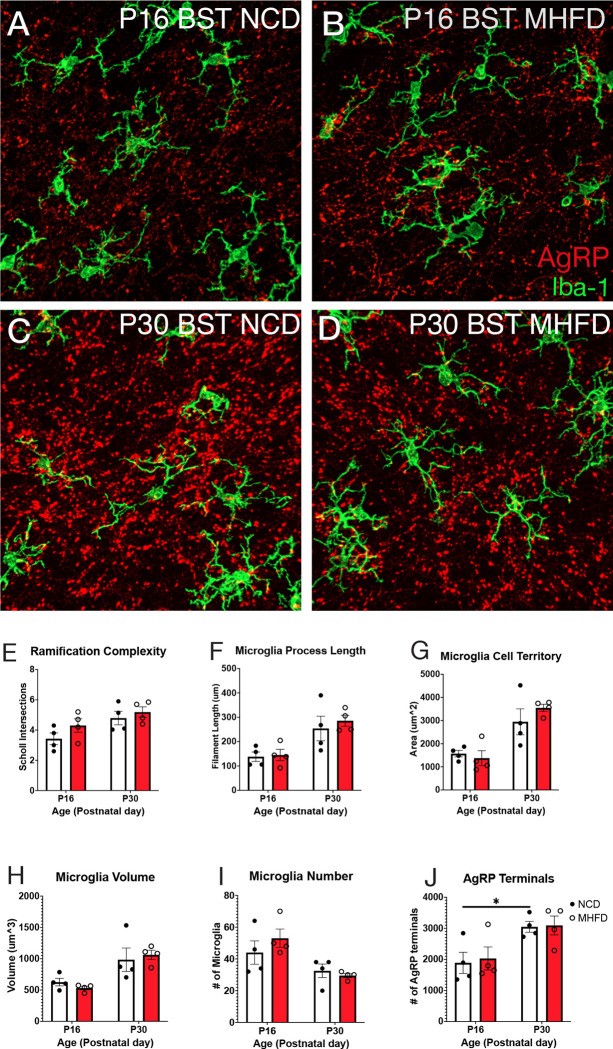
MHFD: Microglial morphology in the BST. Maximum projection images of microglial cells (green) and labeled AgRP terminals (red) in the BST of mice at P16 (A, B) or P30 (C,D) that were either exposed to MHFD (B, D) or NCD (A, C). Graphical comparisons between groups to show that microglial ramification complexity (E) remained the same, regardless of age or diet. Microglial cell territory (F), cell volume (G) and process length (H) did not significantly change between P16 and P30 and were not changed as a result of diet. The density of microglia in the BST decreased between P16 and P30, irrespective of diet (I). The density of AgRP terminals increased between P16 and P30, but there was no effect of maternal diet (J). Bars represent the mean ± SEM and each point represents one animal. *P<.05. Abbreviations: BST, bed nucleus of the stria terminalis; MHFD, maternal high fat diet during lactation; NCD, Normal Chow Diet.

The ARH houses the cell bodies of AgRP neurons, and their number is established primarily during prenatal development (Ishii & Bouret 2012). We evaluated microglia morphology in the ARH of postnatal mice by using the same 3D modeling pipeline shown in Figure 1. In sharp contrast to our findings in the PVH, the number and size of microglia in the ARH were not significantly different in NCD and MHFD offspring at P16 (Figure 2A, B; 2E, F). By P30, there was a 67% increase in process length compared to their P16 counterparts, but no significant difference in microglial morphology in the ARH detected between treatment groups (Figure A–I). The volume of microglial cells, as well as the spatial territory they occupy, also increased between P16 and P30 (Figure 2G–H), but the number of microglia visualized was reduced by nearly half (Figure 2I). We also measured numbers of AgRP neuronal cell bodies in the ARH in brains derived from NCD and MHFD offspring and confirmed that the number of AgRP neurons in the ARH are also resistant to MHFD exposure (Figure 2J). In addition, we evaluated microglia and AgRP terminals in the anterolateral part of the BST, an extrahypothalamic target of AgRP neurons innervated during the lactational period (Bouret, Draper and Simerly, 2004a; Cansell et al., 2012; Barbier et al., 2021). As was found for the ARH, neither the number nor size of microglia in the BST were significantly different between NCD and MHFD offspring at P16 or P30 (Figure 3A–D; 3E–I). Similarly, the density of AgRP terminals in the same region of interest was not affected by MHFD exposure (Figure 3J). By P30 the number of AgRP terminals in the BST increased by 61% compared to their P16 counterparts (Figure 3J). Taken together, these data suggest that microglia in the ARH and BST are resistant to the increases in cellular complexity that occur in the PVH of MHFD offspring, suggesting a notable degree of spatial specificity in the role of hypothalamic microglia during postnatal development.

### 2.3 Microglia are required for changes in AgRP terminal density in PVH and body weight associated with MHFD exposure

**Figure 4.**
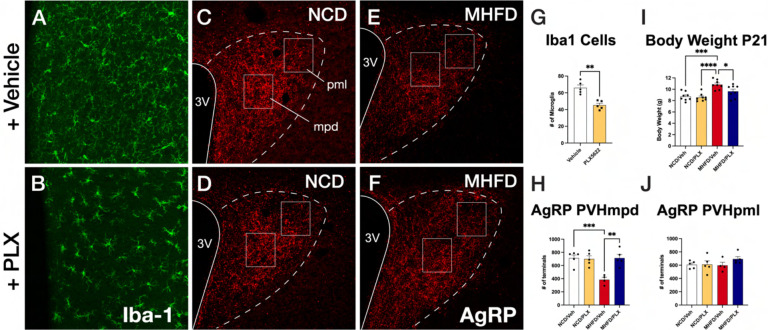
Microglial depletion during lactation period. Images of microglial cells (green) to assess microglia number (A,B). Maximum projection images of labeled AgRP terminals (red) to assess AgRP terminal density in distinct compartments of the PVH at P55 long after the period of daily injections (C-F). Graphical comparison between groups to show that daily postnatal PLX5622 injections caused a 33% decrease in microglia in the PVH (G). Maximum projections of confocal images to illustrate the density of AgRP labeling in the PVH of NCD offspring (C,D) and MHFD offspring (E,F). (G-J) Graphical comparison to illustrate the effects of postnatal PLX5622 treatments on microglia in the PVH (G), body weight (I) and the density of AgRP terminals in the PVHmpd (H) and PVHpml (J). Bars represent the mean ± SEM and each point represents one animal. *P<.05, **P<.005, ***P<.0005. Abbreviations: AgRP, agouti-related peptide; CSF1R, Colony-Stimulating Factor 1 Receptor; MHFD, maternal high fat diet during lactation; MPD, medial parvocellular compartment of the PVH; PML, posterior magnocellular compartment of the PVH; PVH, paraventricular nucleus of the hypothalamus.

To determine if microglia are required for the observed changes in AgRP inputs to the PVH of MHFD offspring, we depleted microglia during the lactation period. The colony-stimulating factor-1 receptor inhibitor (CSF1R) PLX5622 was administered daily via intraperitoneal injections between P4-P21. These postnatal treatments resulted in a significant decrease in microglia in the PVH at the time of weaning (Figure 4A–B; Figure 4G) that appeared to be widespread throughout the rostral forebrain. Notably, the PLX5622 treatments blocked the reduction in AgRP fiber density observed in the medial dorsal parvicellular part of the PVH (PVHmpd) of vehicle-treated MHFD offspring to a level that was comparable to that of NCD offspring (Figure 4C–F; quantified in 4H). In contrast, MHFD exposure did not affect the density of AgRP fibers in the lateral posterior magnocellular compartment of the PVH (PVHpml) and no significant difference in AgRP fiber density was detected between MHFD offspring treated with either PLX or vehicle (Figure 4C–F; 4J), suggesting target specificity for the microglia mediated effects on development of AgRP inputs to the PVHmpd.

Depletion of microglia also appeared to protect against the increase in body weight normally observed in MHFD mice. In keeping with previously published results (Vogt et al. 2014), the weight of vehicle treated MHFD animals was significantly greater (24%), compared with that of NCD animals (Figure 4I). However, MHFD mice treated with PLX5622 during lactation exhibited significantly lower weights at P21 compared to those of vehicle-treated MHFD animals. Taken together, these findings suggest that microglia mediate target-specific effects of MHFD exposure on the innervation of the PVH by AgRP neurons, and that microglia play a role in mediating the effects of MHFD on body weight.

### 2.4 Engulfment of AgRP terminals by microglia in the PVH and the ARH

**Figure 5.**
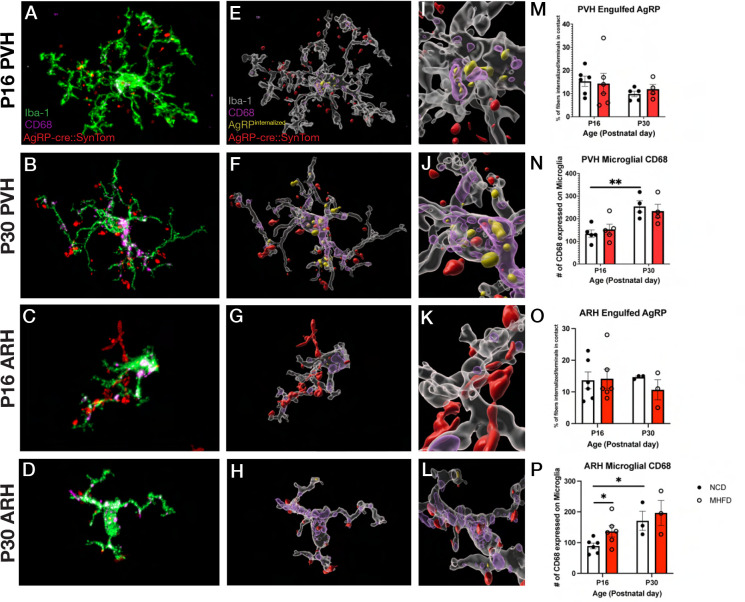
Microglial interaction with AgRP axon terminals in the PVH and ARH. (A-D) Maximum projection images of microglial cells (green) labeled AgRP terminals (red), and CD68 (lysosomal associated membrane protein and phagocytic capacity marker, pink). (E-H) Digital 3D reconstructions shown in (A-D) after application of a digital zoom to more clearly illustrate engulfment of labeled AgRP terminals by microglia (I-L). (M-P) Graphical comparisons between groups to illustrate the effects of age and MHFD exposure on CD68 expression and AgRP terminal engulfment. Bars represent the mean ± SEM and each point represents one animal. *P<.05, **P<.005. Abbreviations: AgRP, agouti-related peptide; ARH, arcuate nucleus or the hypothalamus; CD68, Cluster of Differentiation 68; MHFD, maternal high fat diet during lactation; PVH, paraventricular nucleus of the hypothalamus.

Microglia are thought to impact development of neuronal connections through an active engulfment mechanism and the lysosomal associated membrane protein CD68 has been implicated in this process. Here, we used immunohistochemistry to visualize the presence of in Iba1-labeled microglia in mice with genetically targeted labeling of AgRP terminals. Many apparent contacts between microglial processes and AgRP terminals in the PVH and ARH were observed at P16 and P30, including internalized AgRP terminals (Figure 5A–L). However, the extent of internalization did not appear to be influenced by MHFD exposure; there were no significant differences between internalized AgRP terminals in MHFD and NCD offspring at P16, in either the PVH or ARH (Figure 5M, O). Similarly, we did not detect a statistically significant difference in microglial CD68 levels in the PVH between diet groups at P30 (Figure 5N). Consistent with previous reports, (Wong et al. 2005; Hart et al. 2012) the density of CD68 labeled profiles nearly doubled in the PVH between P16 and P30 (Figure 5N), as microglia become more phagocytic with age. CD68 staining also increased in the ARH between P16 and P30 (Figure 5P), supporting the notion that microglia increase their phagocytic capacity with age. Nevertheless, our analysis demonstrates that microglia interact directly with AgRP terminals, with clear evidence of engulfment. MHFD exposure does not appear to promote microglia-mediated engulfment, at least not in the specific PVH and ARH domains examined.

## 3 Discussion

It is well established that microglia are responsive to HFD exposure in adult rodents (Thaler et al., 2012; Morari et al., 2014; Valdearcos et al., 2014; Baufeld et al., 2016; Valdearcos et al., 2017) and multiple lines of evidence support an important role for microglia in mediating key aspects of neural circuit development (Checchin et al., 2006; Hoshiko et al. 2012; Li et al., 2012; Hagemeyer et al., 2017). Here we demonstrate that microglia are required for significant elevations in body weight that emerge from postnatal exposure to HFD and are associated with a sustained decrease in the density of afferents from AgRP neurons to the PVH in offspring. Exposure to MHFD caused distinct morphological changes to microglia that are consistent with enhanced activity, which were observed in the PVH, but not the ARH or BST. Moreover, the morphological changes to microglia observed appear to be primarily limited to the critical period for development of AgRP inputs to PVH neurons in MHFD offspring. Although our results demonstrate that microglia engage in engulfment of AgRP terminals in the PVH during development, synaptic pruning by microglia does not appear to represent the cellular mechanism mediating the effects of MHFD exposure on innervation of the PVH by AgRP neurons.

### 3.1 A Role for Microglia in Mediating Body Weight Changes Observed in MHFD Offspring

Maternal high fat diet exposure during a period that extends across both gestation and lactation causes offspring to have increased body weight, fat content and susceptibility to diet-induced obesity (Samuelsson et al., 2008; Masuyama et al., 2014; Gomes et al., 2018). In contrast to the more variable impact of maternal HFD exposure just during gestation, limiting HFD exposure to the postnatal period, which corresponds to lactation, when mice derive their nutrition primarily from milk, reliably predisposes adult mice to obesity. Feeding pregnant dams HFD during the week prior to parturition leads to either increased body weight in offspring (Akhaphong et al., 2021) or no significant effect (Sun et al, 2012; Vogt et al., 2014), suggesting that MHFD plays a dominant role in specifying metabolic phenotype later in life (Sun et al., 2012; Vogt et al. 2014). In the present study, global depletion of microglia with the CSF1R inhibitor PLX5622 blocked the ability of MHFD to increase body weight in mice at P21, indicating that microglia may mediate metabolic changes caused by MHFD exposure that are apparent as early as weaning. A previous study that used intragastric administration of PLX3397 to neonatal mice reported enhanced food intake in the offspring during lactation (Sun et al., 2023). Notably, these mice were not derived from MHFD dams. Microglial depletion with PLX5622 in adult mice was found to mitigate the effects of HFD exposure (Rosin et al., 2018) and our results indicate that microglia may function similarly in offspring during postnatal life to effect changes in body weight, even if the maternal HFD exposure is restricted to the lactation period. Further studies are required to define the long-term metabolic profile resulting from developmental microglial manipulations. However, given the abundant literature on sustained impact of MHFD on metabolic phenotype, enduring disruptions are likely. It should be noted that PLX5622 treatment is not spatially limited to the PVH or ARH, leaving open the possibility that the effects of microglial depletion on body weight occur outside of these nuclei, or are due to collective activation of microglia in multiple components of feeding circuitry (Green, Crasper and Hohsfield, 2020). Localization of the specific site of action for microglial specification of mature body weight during development will require utilization of specific markers for hypothalamic microglia that account for regional and phenotypic heterogeneity, perhaps through intersectional genetic methods and combinatorial pharmacology (Hammond et al., 2019; Kim et al., 2021).

### 3.2 MHFD Induces Spatially Limited Changes in Microglial Morphology

Morphological changes in microglia have been reported in response to a variety of environmental exposures. In the hypothalamus, adult mice fed a high fat diet show both proliferation and changes in process length and complexity (Thaler et al., 2012; Valdearcos et al, 2017). In our studies, MHFD exposure caused a marked increase in the overall size of microglia in the PVH related to increases in both the length and branching complexity of Iba-1-stained cellular processes. This increase in the territory occupied by microglia in the PVH was not accompanied by an increase in microglial number, nor were numbers of microglia affected in the PVH by MHFD exposure. However, in contrast to the PVH, changes in microglial morphology were not observed in the ARH in response to MHFD, although we did observe an overall increase in process length in the ARH between P16 and P30 of both MHFD and NCD mice. This finding is consistent with previously published reports on microglial maturation (Sun et al., 2023). The spatially restricted enhancement of microglial activation in the PVH resulting from MHFD exposure appears to contribute to an expansion of parenchymal territory surveilled by PVH microglia, as reflected in the volume measurements accomplished with geometrical modeling of process length and complexity. This interpretation is supported by *in vitro* and *in vivo* observations of enhanced process extension and increased neuronal interactions resulting from inflammatory activation of microglia (Wake et al., 2009; Schafer et al., 2013; Dissing-Olesen et al., 2014; Stowell et al., 2018; Bolton et al., 2022).

As in the ARH, we did not observe comparable changes in microglial morphology in the BST, an extrahypothalamic target of AgRP neurons innervated during the lactation period (Cansell et al., 2012; Barbier et al., 2021). These observations underscore the remarkable molecular heterogeneity of microglial phenotypes that appear to occupy various hypothalamic niches during development, and which may have equally diverse developmental roles and responses to environmental signals (Bilbo and Schwarz, 2012; Frost and Schafer, 2016; Li and Barres, 2018; Ngozi and Bolton, 2022). Moreover, the observed morphological changes in the PVH caused by MHFD appear to be transient as there no significant differences in microglial processes by P30, and the density of microglial cells in the PVH was significantly reduced in the older mice, suggesting a decline in overall activity. Temporal alignment of these morphological events with the critical period for development of AgRP projections to the PVH suggests a possible role for microglia linking nutrition with specification of axonal targeting (Kamitakahara et al., 2018).

### 3.3 Microglia Mediate Impaired Innervation of the PVH by AgRP Neurons

AgRP neuronal projections develop primarily during the first two weeks of life, which corresponds to a critical period for the neurotrophic action of leptin on axonal outgrowth and targeting of AgRP inputs to distinct components of the PVH (Simerly, 2008; Elson and Simerly, 2015). Exposure to MHFD during this critical developmental window permanently impairs AgRP projections to the PVH, DMH and LH (Vogt et al., 2014) that are associated with increased body weight in adulthood. Here we confirm that MHFD impairs innervation of the PVH, and this defect was apparent as early as P16. The PVH is innervated by AgRP neurons between P8 and P10 (Bouret, Draper and Simerly, 2004a). We found that depletion of microglia with PLX5622 between P4 and P21, a period that aligns not only with AgRP innervation of the PVH but also with maximum changes in microglial morphology, blocked the effects of MHFD on AgRP terminals in the PVH. However, this partial rescue of innervation appeared to be limited to the PVHmpd, suggesting that there is a profound regional specialization in the activity of microglia in the PVH. We did not observe a change in AgRP innervation of the BST in MHFD offspring that mirrored the changes seen in the PVH of the same animals, further supporting the conclusion that microglia display significant spatial heterogeneity in mediating site-specific alterations in AgRP axon targeting.

Microglia are known to impact a variety of developmental events, including alterations in cell number through programmed cell death or neurogenesis, as well as axonal targeting and remodeling of neural circuits (Frost and Schafer, 2016; Li and Barres 2018). It is unlikely that the impaired innervation of the PVH observed in MHFD offspring is due to a reduction in the number of AgRP neurons in the ARH (Vogt et al, 2014; Valdearcos et al., 2017). Sun and colleagues reported that microglial depletion during postnatal life actually increases numbers of AgRP neurons, as well as enhances local densities of AgRP fibers in the ARH, possibly through enhanced formation of perineuronal nets (Sun et al., 2023). However, changes in AgRP targets outside of the ARH were not assessed. Depletion of microglia during gestation causes a significant decrease in the number of proopiomelanocortin (POMC) neurons in the ARH and leads to an acceleration of weight gain (Rosin et al., 2018), consistent with neurogenesis of ARH neurons occurring in mid gestation and being susceptible to nutritional impacts during embryonic life (Ishii and Bouret, 2012; Elson and Simerly, 2015). Interestingly, genetic deletion of leptin receptors from myeloid cells reduced numbers of POMC neurons in the ARH, suppressed POMC innervation of the PVH, and decreased microglial process complexity (Gao et al., 2017). Taken together, these results suggest that leptin signaling in microglia may act at the level of the ARH to promote growth of AgRP projections to the PVH, while MHFD activates microglia in the PVH to specify patterns of AgRP afferents that are not only regionally specific, but also target discrete domains of the PVH. However, whether microglia inhibit synaptogenesis or are involved in synaptic refinement through an alternative regressive mechanism will require further investigation.

### 3.4 Microglia in PVH Participate in Synaptic Pruning

Microglia play an important role in synaptic pruning, a process whereby synapses that form early in development are eliminated as others are strengthened and maintained (Katz and Shatz, 1996; Sanes and Lichtman, 1999; Frost and Schafer 2016; Li and Barres, 2018). Although this process has been studied most extensively in somatosensory cortex (Miyamoto et al., 2016), hippocampus (Paolicelli et al., 2011; Wang et al. 2020) and the visual system (Tremblay et al., 2010; Schafer et al., 2012), there is clear evidence for involvement of microglia in synaptic pruning of immunolabeled glutamatergic terminals associated with corticotropin releasing hormone neurons in the PVH (Bolton et al., 2022). In the present study, we used AgRP axonal labeling to provide evidence that microglia in the PVH participate in synaptic pruning of AgRP/GABAergic synapses during the critical period for PVH innervation, and when PVH microglia exhibit high levels of process extension. The lysosomal marker CD68 was colocalized with internalized AgRP terminals in PVH microglia, and although elevated at P30, there were no differences between offspring of MHFD and NCD dams. Additionally, we did not find a significant difference between the density of engulfed AgRP terminals in the PVH of MHFD offspring at either P16 or P30. However, enhanced engulfment of AgRP terminals in MHFD offspring may occur at a later point in development. It is also possible that the synaptophysin-tdTomato axonal label may have been lost from pruned synapses during engulfment. A more probable interpretation is that PVH microglia participate in synaptic refinement through other cellular mechanisms, including microglial release of secreted factors such as IL6 (Kim, Copperi, and Diano, 2024) or microglial derived BDNF (Parkhurst et al., 2013). Additional investigation is required to resolve this question.

## 4 Conclusions

MHFD causes elevated levels of saturated carbohydrates and fats in milk (Gorski et al., 2006; Vogt et al., 2014; Calvo-Lerma et al., 2022). The resulting overnutrition resulting from exposure to this enhanced diet is thought to underly the propensity towards obesity observed in offspring later in life (Skowronski, Leibel and Leduc, 2023). Although microglia are likely mediators of multiple neurobiological events influencing how hypothalamic circuits function during regulation of energy balance, the precise signaling mechanisms remain ill defined. There may be common molecular mechanisms underlying the effects of HFD exposure on microglial activation in adults and those occurring during postnatal development, but how these signaling events exert a lasting impact on the organization and function of feeding circuitry has not been defined. The results presented here clearly demonstrate an important role for microglia, specifically in the PVH, on sculpting the density of AgRP inputs to the PVH that is not only spatially restricted, but also aligned temporally with synaptogenesis in the PVH. Furthermore, PVH microglia clearly interact directly with AgRP afferent axons during this critical period and appear to be refined through engulfment by microglia. However, synaptic pruning does not appear to be sufficient to affect the significant reduction in AgRP innervation of PVH neurons observed following MHFD exposure, suggesting involvement of additional microglial signaling mechanisms that are not only important for specifying patterns of innervation of the PVH by AgRP neurons, but may also contribute more broadly to developmental programming of metabolic phenotype.

## 5 Materials and Methods

### 5.2 Animal Care

Transgenic mice expressing IRES-Cre recombinase under the control of the AgRP promoter, AgRP-Cre mice (AgRP-IRES-Cre Stock no: 012899; MGI:J:140858) and mice expressing the cre-dependent fluorescent reporter synaptophysin-tdTomato (SynTom mice; Ai34D-Rosa-CAG-LSL-Synaptophysin-tdTomato-WPRE; stock number: 012570; MGI:J:170755) were obtained from the Jackson Laboratory (Bar Harbor, ME) and maintained in our colony at Vanderbilt University. Wild-type C57BL/6J mice (stock number: 000664) were also obtained from The Jackson Laboratory. To visualize AgRP inputs, AgRP-Cre mice were crossed with SynTom mice to generate AgRP-Cre::SynTom mice, as described previously (Biddinger, et.al., 2020).

All animal care and experimental procedures were performed in accordance with the guidelines of the National Institutes of Health and the Institutional Care and Use Committee of Vanderbilt University. Mice were housed at 22°C on a 12:12 h light:dark cycle (lights on at 6:00 a.m.: lights off at 6:00 p.m.). Mice were provided ad libitum access to a standard chow diet (PicoLab Rodent Diet 20 #5053), unless otherwise specified.

To generate offspring of dams exposed to HFD during lactation (MHFD) mice had ad libitum access to normal chow (PicoLab Rodent Diet, 5053LabDiet: 25% protein; 62% carbohydrates; 13% fat; 4 kcal/g energy density) during mating. On the first postnatal day (P1) all litters were adjusted to 7 pups to normalize nutrition and dams were switched to either high fat diet (HFD) (Research Diets D12451: 20% protein; 35% carbohydrate; 45% fat; 4.7 kcal/g energy density) or kept on normal chow diet (NCD). The dams remained on either HFD or NCD through lactation, and at weaning the offspring were switched to a normal chow diet, regardless of lactation diet condition.

### 5.3 PLX5622 Microglia Depletion

To reduce microglia during postnatal development (P4-P21) mice were treated with PLX5622, a colony stimulating factor 1 receptor (CSF1R) inhibitor or with DMSO vehicle via intraperitoneal injection (Riquier et al. 2020). Briefly, PLX5622 hemifurate solid (Cat. #HY114153A MedChemExpress, Monmouth Junction, NJ, USA) was suspended in dimethyl sulfoxide at a concentration of 172 mg/ml. The injection working solution was prepared to include 20% Kolliphor RH40 diluted in PBS, which resulted in doses with a 6.5mg/ml PLX5622 concentration and injection concentration of 15 mg/kg. IP injections were given once daily from P4 to P21 and injection dose was determined by each animal’s body weight at time of injection.

### 5.4 Immunohistochemistry

Mice were perfused at P16 and P30 and processed for immunofluorescence by using an antibody to Iba-1 (1:2000; FUJIFILM Wako Pure Chemicals, Osaka, Japan) to visualize microglia and an antibody to CD68 (1:500; Abcam, Cambridge, MA, USA) to assess phagocytic capacity of the microglia. Mice were first anesthetized with tribromoethanol (TBE) and then perfused transcardially with cold 0.9% saline, followed by cold fixative (4% paraformaldehyde in borate buffer, pH 9.5) for 10 minutes. Brains were then removed from skull and postfixed in the same fixative overnight. Brains were then cryoprotected overnight in a 20% sucrose solution before being frozen in optimal temperature cutting compound (Sakura Finetek Inc., Torrance, CA) and sectioned on a freezing stage sliding microtome at 30μm. Free-floating brain sections were rinsed in KPBS and then incubated in blocking buffer containing 2% normal donkey serum and 0.3% Triton-X overnight at 4°C. Sections were transferred to primary antibody incubation for 48hr with rabbit anti-Iba-1 antibody and rat antiCD68 antibody. Following primary antibody incubation, sections were rinsed several times in KPBS, incubated for an hour at room temperature in blocking buffer containing secondary antibodies against rabbit and rat (raised in donkey) conjugated with Alexa-Fluor fluorochromes (1:500; Life Technologies, Carlsbad, CA, USA), mounted onto charged microscope slides and coverslipped using ProLong antifade mounting medium (Life Technologies, Carlsbad, CA, USA).

### 5.5 Image Acquisition and Analysis

Sections through the PVH, ARH, and BST were examined on a laser scanning confocal microscope (Zeiss 800) and cytoarchitectonic features of the nuclei were visualized with Hoescht 33342, and used to define matching regions of interest (ROI) for quantitative analysis. Confocal image stacks were collected for each ROI through the entire thickness of the region at a frequency of 0.1μm using the 40x objective. Imaris visualization software (Bitplane V9.5, Salisbury Cove, ME, USA) was used to create 3D reconstructions of each multichannel set of images.

3D reconstructions of microglia based on Iba-1 immunofluorescence were made using made using Imaris (Bitplane, v9.5). Profiles were skeletonized automatically by using the Filaments tool in order to quantify changes in microglia structure. Sholl analysis (Geoffry et al., 2014), was performed on the skeletonized structures to determine complexity of branching of microglial processes. Briefly, 3D concentric spheres are drawn around the microglia skeleton and each contact point between microglial process and sphere is counted as 1 level of branching. The higher the number of contacts, the higher complexity of branching in the microglia. 3D reconstructions of the microglia were also evaluated for length of processes as well as cell volume and the area that is occupied. To evaluate the 3D space occupied by each microglial cell, a polyhedron was drawn around the microglia by using the built-in Convex Hull function under Filaments, which is used to calculate volume of the polyhedron automatically, accounting for the 3D space around the microglia.

In order to assess cellular interactions between AgRP terminals and microglia, the 3D renderings were used to determine level of contact by using a MATLAB script to automate the analysis. Both channels were subjected to background subtraction and Gaussian filtering. The automatic threshold calculated was based on k-means statistical methods and was used in the majority of analyses. The AgRP terminals were reconstructed as ‘‘spots’’ of 0.8 mm diameter (corresponding to the largest measured size) and their total number was automatically calculated. Briefly, the automatic detection algorithm applies a 3D Mexican hat filter using the spot size and then locates the spot centroid at the local maxima of the filtered image. The number of spots located at no more than 1μm from the microglia surface was automatically determined and indicated as a contact point. Next, spots that were determined to be less than 0.5μm away from internal microglial surfaces were determined to be internalized by the microglia and counted as engulfed. CD68 levels were determined using automated analysis to create 3D renderings and their volume computed.

### 5.6 Statistical Analyses

Data are presented as the mean values ± SEM. Descriptive statistics and unpaired t-tests were used to compute group differences using GraphPad Prism software (GraphPad Software, San Diego, CA). P-value less than 0 .05 were considered significant.

## Figures and Tables

**Key Resources Table:** 

Reagent type or resource	Designation	Source or reference	Identifiers	Additional information
Genetic reagent (*M. musculus*)	*AgRP*-IRES-Cre	Jackson Laboratory	Stock #: 012899 RRID: IMSR_JAX:012899	MGI ID: J:140858
Genetic reagent (*M. musculus*)	Synaptophysin-tdTomato	Jackson Laboratory	Stock #: 012570 RRID: IMSR_JAX:012570	MGI ID: J:170755
Antibody	Rabbit polyclonal anti-Iba1	FUJIFILM Wako Shibayagi	Cat. #: 019-19741 RRID: AB_ 839504	IHC (1:2000)
Antibody	Rat monoclonal anti-CD68 [FA-11]	Abcam	Cat. #: ab53444 RRID: AB_869007	IHC (1:500)
Antibody	Donkey polyclonal anti-rabbit Alexa Fluor 488	ThermoFisher Scientific	Cat. #: A32790 RRID: AB_2762833	IHC (1:500)
Antibody	Donkey polyclonal anti-rat Alexa Fluor 647	ThermoFisher Scientific	Cat. #: A48272 AB_2893138	IHC (1:500)
Pharmacological Inhibitor	PLX5622 hemifumarate, CSF1R inhibitor	MedChemExpress	Cat. # HY114153A	
Software, Algorithm	Imaris	Bitplane	V9.5	
Software, Algorithm	GraphPad Prism	Prism	Prism 7	

## Significance Statement

Maternal high fat diet exposure results in enhanced risk for negative health outcomes in humans and multiple animal models. Here we demonstrate that microglia are required for changes in body weight and perturbations to hypothalamic circuits caused by maternal high fat diet exposure that is limited to the lactational period. We identified spatially and temporally limited morphological changes to microglia that reflect an enhancement of surveillance activity and align with a critical period of hypothalamic circuit formation. We also identify direct cellular interactions between microglia and developing axons, as well as evidence for synaptic engulfment, although this mechanism does not appear to be responsible for changes to neural patterning caused by maternal high fat diet exposure. Together these findings identify an essential role for microglia in specifying patterns of hypothalamic innervation during development in response to maternal high fat diet exposure, which may contribute to developmental programming of metabolic phenotype.
